# Judging orthodontic treatment complexity

**DOI:** 10.1590/2177-6709.21.1.060-066.oar

**Published:** 2016

**Authors:** Maïté Clijmans, Aly Medhat, An De Geest, Johannes van Gastel, Annelies Kellens, Steffen Fieuws, Guy Willems

**Affiliations:** 1 Orthodontist consultant, KU Leuven & Dentistry, University Hospitals Leuven, Department of Oral Health Sciences - Orthodontics, Leuven, Belgium; 2 Statistical consultant, KU Leuven & Dentistry, University Hospitals Leuven, Department of Oral Health Sciences - Orthodontics, Leuven, Belgium; 3 Professor, Head of Department, Program Director, KU Leuven & Dentistry, University Hospitals Leuven, Department of Oral Health Sciences - Orthodontics, Master and Postgraduate, Leuven, Belgium

**Keywords:** Orthodontic treatment complexity, Orthodontic indices

## Abstract

**Objective:**

The aim of the present study was to investigate possible relations between anticipated overall treatment complexity (AOTC) of an orthodontic case and malocclusion characteristics.

**Methods:**

Two groups of orthodontists (groups A and B) were asked to define perceived treatment complexity (PTC) of orthodontic cases based on 16 characteristics of malocclusion by means of a questionnaire. Each question was answered on a six-point ordinal scale, with one "not applicable" option (score 0). Group A was also asked to give the AOTC of the specific case on a five-point ordinal scale. The index of orthodontic treatment need (IOTN) score of the specific cases as well as the malocclusion characteristics were assessed by one author.

**Results:**

There is a significant relationship between IOTN and AOTC (*p*< 0.0001), 22% of variability is explained by differences in IOTN. Adding objective characteristics of malocclusion to explain AOTC does not significantly increase the explained variability (*p* = 0.086). In judging interobserver agreement, a weighted Kappa of 0.60 for group A and 0.56 for group B was found. The weighted Kappa for agreement in AOTC equals 0.06.

**Conclusion:**

The relation between IOTN and AOTC was found to be significant. Moderate agreement on PTC among observers and a low level agreement regarding AOTC were found in the present study.

## Abstract (pt)

**Objetivo:**

o objetivo do presente estudo foi investigar possíveis relações entre a previsão da complexidade geral do tratamento (AOTC, *Anticipated Overall Treatment Complexity*) de um caso ortodôntico e as características da má oclusão.

**Métodos:**

dois grupos de ortodontistas (Grupo A e Grupo B) foram orientados a definir, por meio de um questionário, sua percepção da complexidade do tratamento (PTC, *Perceived Treatment Complexity*) de casos ortodônticos, com base em 16 características da má oclusão. Cada questão foi respondida em uma escala ordinal de seis pontos, com a opção "não aplicável" (nota 0) disponível. Pediu-se ao Grupo A que também determinasse, em uma escala ordinal de cinco pontos, a AOTC de cada caso. A nota relativa ao índice de necessidade de tratamento ortodôntico (IOTN,*Index of Orthodontic Treatment Need*) de casos específicos e as características das más oclusões foram avaliadas por um dos autores.

**Resultados:**

existe uma relação significativa (*p* < 0,0001) entre o IOTN e a AOTC. Diferenças no IOTN são a causa de 22% da variabilidade. Adicionar características objetivas da má oclusão para explicar a AOTC não aumentou significativamente essa variabilidade mencionada (*p* = 0,086). Ao se avaliar a concordância interexaminadores, foram encontrados valores de Kappa ponderado de 0,60 para o Grupo A e 0,56 para o Grupo B. O valor de Kappa ponderado para a concordância quanto à AOTC foi de 0,06.

**Conclusão:**

a relação encontrada entre o IOTN e a AOTC foi considerada significativa. Verificou-se uma moderada concordância interexaminadores com relação à PTC, e um baixo nível de concordância com relação à AOTC.

## INTRODUCTION

Interest in developing an index of orthodontic treatment complexity has increased over the years, as such an index could be used to assign resources and determine appropriate financial compensation for treatment. Moreover, an index of orthodontic treatment difficulty could be used to increase the esteem of the practicing professionals by recognizing professionals treating complex cases.^1^^,^ 2 Often, the terms "complexity", "difficulty" and "severity" are used in the same context. In Orthodontics, previous authors have suggested that "difficulty" and "complexity" are synonymous and should be defined as a measure of effort and skill, while severity is a measure of how much a malocclusion deviates from the ideal.^3^ We agree and will use this terminology.

Up to the present, no orthodontic treatment complexity index has been developed and used, although several attempts have been made. Daniels and Richmond^3^ developed an index of complexity, outcome and need (ICON) to assess treatment inputs (need), outputs and complexity. ICON has been shown to be a reliable and valid index used to assess orthodontic treatment need.^4^^,^ 5 However, ICON has its limitations. For example, it is heavily weighted for aesthetics, an assessment that is highly subjective, which reduces its objectivity.^2^ The validity of ICON for measuring orthodontic complexity, outcome and treatment improvement has been previously studied.^6^ The results showed that intraexaminer agreement was moderate for complexity, slight for outcome, and poor for degree of improvement, while interexaminer agreement was moderate for complexity and outcome and only fair for degree of improvement. More recently, the index of orthodontic treatment complexity (IOTC) was developed specifically to measure treatment complexity.^2^ IOTC is based on the Peer Assessment Rating index by applying different weighting to each component.^7^ The authors suggest that IOTC shows sufficient promises to warrant further development, but no other publications about it appeared ever since. Given the contradictions of different studies regarding the development of an orthodontic treatment complexity index to evaluate several aspects of orthodontic treatment, further investigation is necessary to unravel the whole issue and take a step closer to the development of an orthodontic treatment complexity index.

Defining orthodontic treatment complexity is modulated by the interaction between factors related to patient's compliance, clinician's skill and experience, and malocclusion characteristics and severity.^3^^,^ 8 ^-^ 11 More specifically, previous studies have reported that there seems to be a correlation between orthodontists' perception of severity of malocclusion characteristics and treatment complexity.^11^^-^ 14 In this study, we focus on the correlation between severity and complexity because only malocclusion characteristics and their severity can be measured more or less objectively before treatment. 

The main objective of the present study, therefore, is to investigate the relation between the objective characteristics of malocclusion and the anticipated overall treatment complexity (AOTC).

## MATERIAL AND METHODS

**Figure 1:**
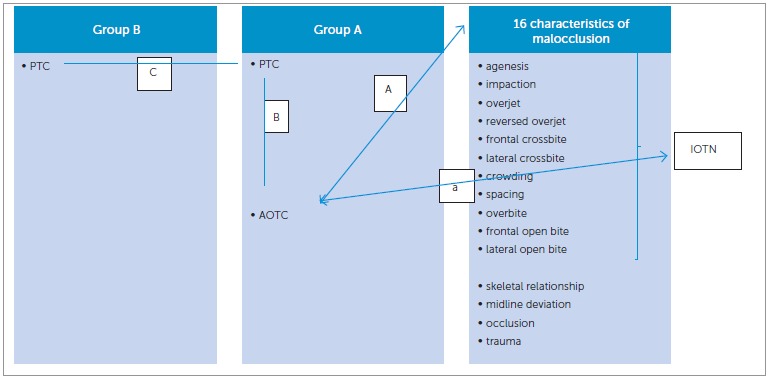
- Overview of investigated relations. A = relation between anticipated overall treatment complexity (judged by group A) and 16 characteristics of malocclusion; a= relation between anticipated overall treatment complexity (judged by group A) and IOTN-score. B= interobserver agreement on perceived treatment complexity and interobserver agreement on anticipated overall treatment complexity (group A) as well as a relation between the perceived orthodontic treatment complexity and anticipated orthodontic treatment complexity. C= interobserver agreement on perceived orthodontic treatment complexity between group A and B.

The study sample consisted of complete orthodontic records of 97 patients: dental casts, lateral cephalometric and panoramic radiographs, and extra- and intraoral photographs taken at the start of orthodontic treatment. All patients received orthodontic treatment at Department of Oral Health Sciences - Orthodontics, KU Leuven & Dentistry, University Hospitals Leuven, between 2005 and 2010. The sample represented a variety of orthodontic malocclusions, all of which had been included among the final examination cases of several graduate students in Orthodontics. Patients with craniofacial anomalies were excluded. The study casts were scored using the index of orthodontic treatment need (IOTN) which determines treatment need based on the dental health condition (Dental Health Component) as well as the aesthetic appearance (Aesthetic Component) of the dentition. In this study, only the Dental Health Component of IOTN was used, which is a 5-grade index that records the dental health need for orthodontic treatment. This was done by one author who had been calibrated in the use of the index.^15^ The same author also noted the 16 characteristics of malocclusion at a different time (Fig 1). These malocclusion characteristics are those of IOTN extended with "skeletal relationship", "midline deviation", "occlusion", and "trauma". Note that the presence of a forced bite can only be assumed, and that crowding and spacing can be summarized as arch length discrepancy (ALD) of the lower or upper jaw. 

In group A (experts), all cases were examined separately by four expert observers who were themselves involved in clinical teaching at university level. Three of them have a PhD in Orthodontics, and more than 15 years of clinical practice, while the fourth observer has been an orthodontic consultant at the university for more than 30 years. These experts were informed about the study protocol. No time limit was set for them.

Subsequently, 20 cases were selected out of the original 97, taking into account the average overall complexity level of the individual cases, as determined by group A, thereby resulting in a fair distribution of easy and difficult cases. A total of 37 nonexpert orthodontists (group B) of a study group were then asked to participate in the present study.

Group A was asked to record their perception of treatment complexity (PTC) of each case based on the severity of the specific malocclusion. For this purpose, a questionnaire was set up with 16 questions, concerning specific malocclusion characteristics, the intention being to evaluate their influence on the rating of treatment complexity. The questionnaire was to be answered on a scale from 1 to 5, with 1 = easy, 2 = mild, 3 = moderate, 4 = difficult, 5 = very difficult. One "not applicable" option (score 0) was added. For each characteristic, the observers were asked the following: "How would you rate treatment complexity of this orthodontic case taking into account this particular characteristic of malocclusion?". The answers to these 16 questions provide an assessment of the PTC for each one of the 16 malocclusion characteristics, as judged by the experts. These observers were also asked to score the AOTC of the orthodontic case as a whole on a five-point ordinal scale, with 1 = easy, 2 = mild, 3 = moderate, 4 = difficult, and 5 = very difficult. This is a more or less subjective estimate. The observers were asked to record their assessments on a scoring sheet which contained information about the age and sex of the individual patients, but no patient identifiers. These expert observers were also asked to suggest a treatment plan for each evaluated case. This was done in order to relate possible differences in PTC to the suggested treatment plan.

A relation between AOTC of an orthodontic case and objective characteristics of malocclusion was screened (Fig 1). More specifically, it was determined whether other objective factors (skeletal relationship, midline deviation, occlusion, and trauma) play a role in anticipating the complexity of an orthodontic case besides those constituting the IOTN. For the same purpose, the relation between the IOTN score and the AOTC was examined (Fig 1). 

Observers of group B (nonexperts) screened the 20 selected cases with the same questionnaire, so as to provide the PTC score for each one of the 16 occlusal characteristics. Some trial cases were discussed in group B before starting the evaluation of the 20 cases in order to get familiar with the study protocol. For group B, treatment plan was suggested in advance and the observers only had to define the PTC of the specific case based on the proposed treatment plan. This was partially due to lack of time, but also to determine if agreement would thereby increase.

Hence, the present study also evaluates the agreement between observers on the PTC, as well as their agreement on the AOTC. The relation between PTC and AOTC was also investigated (Fig 1, relation B) as well as the agreement between the PTC, as judged by four experts versus 37 nonexperts (Fig 1, relation C). 

### Statistics

Mann-Whitney U tests, Kruskal-Wallis tests and Spearman correlations were used to explore relations between IOTN and the characteristics of malocclusion on one hand, and the mean AOTC (mean of the four experts in group A) on the other hand. Linear regression models were used with the AOTC as a dependent variable. In each model, the percentage explained variability was reported. In a first model, the independent variable was IOTN (treated as a categorical variable). In a second model, the independent variables were the characteristics of malocclusion with *p* < 0.10 in invariable analyses. A third model combined these characteristics and the IOTN as independent variables, such that it was verified if the malocclusion characteristics contributed with additional information to the IOTN in explaining the variability in anticipated overall treatment complexity. Weighted Kappa was used to evaluate interobserver agreement of AOTC between the four observers in group A (1-5 score). Systematic differences between observers in the distribution of these scores were verified with signed-rank tests. Kappa and weighted Kappa were used for the anticipated complexity, as based on each one of the 16 malocclusion characteristics (1-5 score and "not applicable"). Spearman correlation was reported for the association between the mean anticipated overall complexity and the mean perceived treatment complexity, as based on each one of the 16 malocclusion characteristics (considering "not applicable" as a zero value). P-values smaller than 0.05 were considered significant. All analyses were performed with the aid of SAS software, version 9.2 of the SAS System for Windows.

## RESULTS

**Figure 2:**
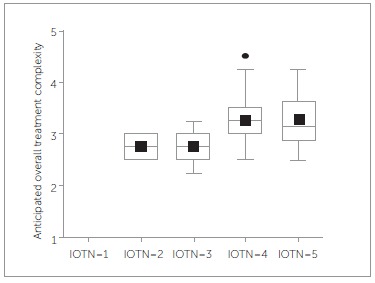
- Relation between anticipated overall treatment complexity and IOTN score.

The mean AOTC of the 97 cases equals 3.2 (SD = 0.5, range: 2.3-4.5). A significant relation was found between IOTN and AOTC (Spearman rho = 0.34, *p* = 0.0007) (Fig 2). 22.24% of variability is explained by differences in IOTN (linear model with IOTN as categorical variable). 

**Figure 3:**
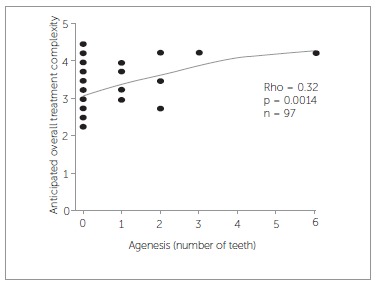
- Relation between anticipated overall treatment complexity and agenesis.

**Figure 4:**
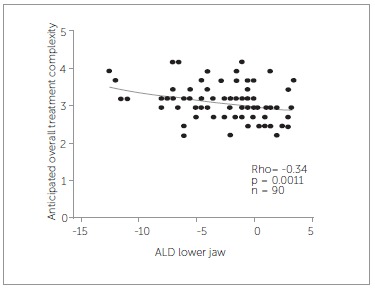
- Relation between anticipated overall treatment complexity and ALD lower jaw.

As for the relation between the 16 characteristics of malocclusion and AOTC, only agenesis (rho = 0.32, *p* = 0.0014) and ALD lower jaw (rho =-0.34,*p* = 0.0011) are significantly related with AOTC (Figs 3 and 4). Therefore, treatment can be anticipated to be more difficult when one or more teeth are absent or with extensive arch length deficiency in the lower jaw. Both the presence of trauma and ALD upper jaw correlate with AOTC, but not significantly. Treatment was anticipated as being more difficult if trauma was present (*p* = 0.061) or with more extensive arch length deficiency in the upper jaw (rho = -0.2, *p* = 0.061).

In a multivariable regression model, these four characteristics (agenesis, ALD lower and upper jaw, and trauma) combined explain 12.9% of variability in AOTC. IOTN, on the other hand, explained 22.4% of variability. When these four characteristics are added to IOTN, the explained variance equals 30.6%, but this increase of 8.2% (compared with 22.4% using only IOTN) due to the inclusion of the set of objective characteristics is not significant (*p* = 0.086). 

Interobserver agreement (Kappa-statistic) of PTC between groups A and B on the subset of 20 cases equals 0.41 (SE = 0.013), which is comparable with interobserver agreement between the experts of group A observed on the total set of 97 cases (k = 0.40, SE = 0.006), as well as with the Kappa of group B based on the same subset of 20 cases (κ = 0.42, SE = 0.001). The weighted Kappa equals 0.61, which is also of the same order as the weighted Kappa on the full set (0.60) and the weighted Kappa for the nonexperts in group B (0.56). Lack of agreement between the four experts can be explained by systematic difference in their scores (Table 1).

**Table 1:** - Systematic difference in scores between four raters in perception of orthodontic treatment complexity (PTC) based on malocclusion characteristics only).

	**Score 1**	**Score 2**	**Score 3**	**Score 4**	**Score 5**
Rater 1	2.0%	47.4%	43.3%	7.2%	0.0%
Rater 2	1.0%	32.0%	54.6%	10.3%	2.1%
Rater 3	0.0%	3.1%	37.1%	42.3%	17.5%
Rater 4	0.0%	6.2%	45.4%	37.1%	11.3%

There is a remarkably low level of agreement between the four observers for AOTC. The probability that two raters agree only equals 0.34, yielding a Kappa of 0.04 (SE = 0.03). The weighted Kappa equals 0.06. An important reason for the lack of agreement is the systematic difference between the four raters in the AOTC (Table 2). The distribution in scores differs significantly between each possible pair of observers (all p-values smaller than 0.01).

**Table 2:** - Systematic difference in scores between four raters in anticipated overall treatment complexity (AOTC).

	**Score 0**	**Score 1**	**Score 2**	**Score 3**	**Score 4**	**Score 5**
Rater 1	60.8%	7.9%	13.6%	11.8%	5.8%	0.1%
Rater 2	59.1%	11.5%	18.2%	9.5%	1.5%	0.2%
Rater 3	60.9%	4.8%	9.3%	8.0%	10.0%	7.0%
Rater 4	59.0%	9.0%	7.0%	13.9%	9.7%	1.5%

**Figure 5:**
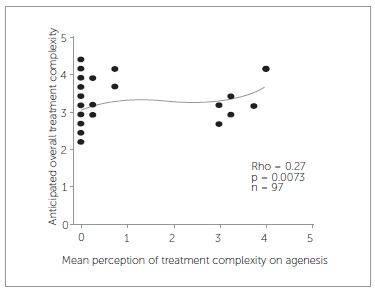
- Relation between anticipated overall treatment complexity and perception of treatment complexity on agenesis.

Exploration of a relation between the PTC and the AOTC reveals significant relations with malocclusion characteristics, such as agenesis (Fig 5), lateral open bite, skeletal relationship and occlusion.

## DISCUSSION

A significant relation between the IOTN-score and the AOTC was found. However, including other objective characteristics of malocclusion (skeletal relationship, midline deviation, occlusion and trauma), besides those constituting the IOTN, does not explain significantly more of the variability, which means they do not contribute to the description of orthodontic treatment complexity. Therefore, including other aspects of malocclusion, such as those suggested in this study, does not significantly contribute to the development of a standardized tool for estimating orthodontic treatment complexity. 

According to the available literature, there indeed seems to be a correlation between the need for orthodontic treatment and treatment complexity. A linear relationship between pre-treatment need based on the ICON index and treatment complexity, as judged on a five-point scale, was found.^3^ A correlation between treatment difficulty and malocclusion severity has been verified in the past.^12^^,^ 14 Bergström et al^9^ have concluded that treatment difficulty was associated with pre-treatment need: the greater the treatment need, the greater the treatment complexity.

The 16 characteristics of malocclusion investigated in the present study explained 30.6% of variance. However, it can reasonably be assumed that complexity of orthodontic treatment is not only due to malocclusion-related aspects, but also to the interaction between patient and practitioner. It has been extensively reported that adequate oral health and optimal compliance are essential for successful orthodontic treatment.^1^ Moreover, the orthodontist's clinical skills and experience contribute significantly to this mission. Finally, the characteristics of a malocclusion and its specific extent add to the complexity of orthodontic treatment. It was reported that the main parameters in determining treatment complexity appear to be pre-treatment age, number of appointments and the initial index of complexity, as well as orthodontic treatment need-score (ICON-score).^1^ These parameters were all found to be significantly associated with judgment of treatment complexity. Cassinelli et al^11^ showed that post-treatment evaluation of treatment difficulty is related to pre-treatment malocclusion severity, among other aspects of treatment, such as patient's age, the number of appointments, changes in treatment plan, oral hygiene, and compliance issues. It seems that aspects other than malocclusion severity are also important in judging orthodontic treatment complexity and that most of these other aspects can only be judged during orthodontic treatment. Treatment complexity is clearly of a multifactorial nature.^1^^,^ 11 ^,^ 16

A moderate agreement on PTC among observers was found in the present study.^17^ However, since a difference in score of one unit can be considered as still acceptable, a high level of agreement was found. On the other hand, there is a remarkably low level of agreement between the four observers regarding AOTC. This is in contrast to the result of a previous study which found a substantial inter-rater agreement for complexity (weighted κ = 0.51) when judging the complexity of orthodontic treatment on a five-point orbital scale.^6^ Since the level of agreement between the four observers is low, a standardized tool for estimating orthodontic treatment complexity seems to be impossible. Apparently, there is more agreement on PTC regarding malocclusion characteristics separately than on the AOTC of an orthodontic case. Asking an expert for his opinion regarding the overall complexity of an orthodontic case does not give the practitioner a better idea of the complexity to be expected, given the low level of agreement between experts. One expert might well rate it differently than another. 

One explanation for the observed differences may be related to the orthodontist's skills and experience, as previously suggested. What might be difficult to treat for one orthodontist can be perceived by another as easy.

Besides the subjectivity in judging treatment complexity, a part of the disagreement between observers is clearly due to a systematic difference between observers for the AOTC as well as for PTC based on malocclusion characteristics only (PTC) (Table 1 and Table 2).

Moreover, the presence of missing scores within the results can explain a part of the noticed disagreement. Although the four expert observers completed all the questions, there were 15 missing scores (out of 592) among the 37 group B orthodontists. This could have been due to lack of time.

Furthermore, the disagreement found in the PTC based on malocclusion characteristics can only be related to orthodontic treatment plan. Some amount of difference in scoring between the four observers of group A could be attributed to their individually proposed treatment plan. Although the observers in group A were not given a treatment plan beforehand, the suggested orthodontic treatment for all cases did not vary a great deal among the four observers. The most common difference in the suggested orthodontic treatment plan, for example, were cases for which one observer would extract two teeth in the upper jaw while another observer would use a headgear appliance to correct the malocclusion. This could lead to some level of disagreement in scoring the complexity of orthodontic treatment.

Comparing the agreement between both groups, a relatively good correlation in qualifying the PTC based on malocclusion characteristics is seen. However, the small difference in the Kappa score between both groups can be explained by the presence of missing scores within group B. Although when defining the PTC in group B calibration was performed in advance of the study, we found that this calibration did not lead to a higher level of agreement within this group.

It is noteworthy that this study has examined anticipated treatment complexity, a priori treatment complexity of an orthodontic case, which is not necessarily the same as the experienced complexity/difficulty during treatment. The latter can be a subject for further investigation. 

## CONCLUSION

A significant, yet modest, relation between the IOTN-score and the AOTC was found. Including other characteristics of malocclusion (skeletal relationship, midline deviation, occlusion and trauma) does not significantly explain more of the variability. A moderate agreement on PTC (based on the 16 characteristics of malocclusion) among the observers was found in the present study. However, there is a remarkably low level of agreement between the four expert observers regarding AOTC.
